# The Assessment of Marcaine Versus Meperidine for Spinal Anesthesia in Anorectal Surgery: A Randomized Clinical Trial

**DOI:** 10.5812/aapm-136871

**Published:** 2023-09-08

**Authors:** Mehran Rezvani Habibabadi, Masumeh Safaee, Ali Rezaei

**Affiliations:** 1 Department of Anesthesiology, School of Medicine, Isfahan University of Medical Sciences, Isfahan, Iran; 2 Kashani Hospital, Isfahan University of Medical Sciences, Isfahan, Iran; 3 Department of General Surgery, School of Medicine, Isfahan University of Medical Sciences, Isfahan, Iran

**Keywords:** Anorectal Fissure, Manometry, Pain, Marcaine, Meperidine

## Abstract

**Background:**

Spinal anesthesia (SA) for the surgical management of chronic anal fissures is favored by surgeons as it provides an early return to daily activities; however, the agents applied for SA to achieve the best outcomes with minimized adverse effects are a matter of debate.

**Objectives:**

This study aimed to assess the utility of Marcaine versus meperidine for SA induction of anoderm surgery.

**Methods:**

This randomized clinical trial (RCT) was conducted on 138 patients with chronic anal fissures who were candidates for surgical management in 2020. The patients were randomly assigned to two groups of SA using 2.5 mL of hyperbaric Marcaine 0.5% (n = 69) or 1 mg/kg of meperidine (n = 69). Pain severity (measured via Numerical Rating Scale (NRS)), anal sphincter tone manometry (measured at baseline and the end of the sphincterotomy), and drug-related adverse effects were compared between the groups.

**Results:**

Both agents led to significant pain relief within 24 hours after SA (P < 0.05); nevertheless, pain severity was remarkably lower in meperidine-treated patients in different measurements performed during the first 24 hours after SA (P < 0.05). The sphincteric tone significantly decreased in both groups (P < 0.001), while the postoperative tone was significantly less in the Marcaine-treated patients (65.22 ± 3.02 versus 46.04 ± 1.97, P < 0.001). The two groups did not differ regarding the adverse effects (P > 0.05).

**Conclusions:**

Meperidine for SA in anal fissure surgical management was relatively superior to Marcaine, as postoperative pain control was remarkably better achieved with meperidine. However, anal sphincter tone reached a normal range in Marcaine-treated cases, and the average tone in those anesthetized with meperidine was slightly above the normal limits.

## 1. Background

An anal fissure that occurs with a linear ulcer in the squamous epithelium of the anus, distal to the dentate line, is one of the most common, annoying, and painful anorectal diseases inflicting a large population worldwide (1). This condition can become chronic and is characterized by indurated edges, visible fibers of the internal anal sphincter at the base of the fissure, and a sentinel polyp or tag at the distal end of the fissure (2).

Numerous conservative options for anal fissure management, including dietary habit changes and local medications, have been proposed; however, due to the chronic nature and high recurrence rate of anal fissures, the surgical approach has been favored after several failures in nonoperative medical management (3).

Considering the severity of the pain caused by the fissure pressure, the choice of anesthesia has long been a matter of debate. Besides, severe postoperative pain can lead to surgery-related complications, including the lack of discharge of respiratory secretions, ileus, urinary retention, and prolonged bed rest. Accordingly, some older studies recommended general anesthesia (GA), while others preferred spinal anesthesia (SA) (4-6). Nowadays, SA is more favored as the patients can return to their activities earlier; still, the agents administered to achieve an appropriate SA with minimal side effects remain challenging (7).

Marcaine is one the oldest anesthetics used for SA for years ago. The most significant benefit of this agent is its short-acting nature which can lead to a quicker recovery. Nevertheless, as Marcaine blocks the sympathetic neural system, it has adverse effects such as hypotension, bradycardia, and transient neurologic symptoms (TNS). These conditions have questioned the routine use of this agent for SA (8-10).

Meperidine is an opioid agent that has attracted anesthesiologists’ attention due to its structural similarity to anesthetics agents (11). The pain relief potency of meperidine is one-tenth of morphine, and it acts by inhibiting the microfibers of afferent neurons in the posterior spinal column. Its interaction with calcium canals restricts neurotransmitters’ release and blocks pain signals. The intrathecal administration of meperidine led to acceptable anesthesia with negligible adverse effects such as nausea, vomiting, itching, and hypotension (12, 13).

## 2. Objectives

Due to the early mobilization requirement after anal fissure surgery and the lack of adequate knowledge about the best agent for SA for the surgical management of this condition, the current study aimed to compare Marcaine with meperidine.

## 3. Methods

This randomized clinical trial (RCT) was conducted on 138 patients with chronic anal fissures who were candidates for surgical management. These patients were admitted to Khorshid and Ayatollah Kashani hospitals affiliated with Isfahan University of Medical Sciences from January to December 2020.

The study protocol that met the tenets of the Declaration of Helsinki was proposed to and approved by the Ethics Committee of Isfahan University of Medical Sciences (IR.MUI.RESEARCH.REC.1397.105). The study was also registered in the Iranian Registry of Clinical Trials (IRCT20180921041078N1). The patients were briefed about the study; they were assured about the confidentiality of their information and signed a written consent form.

Patients aged over 18 years suffering from chronic anal fissures who had not responded to conservative treatments for at least six months and complained of severe pain (≥ 6 based on the Numerical Rating Scale (NRS)) (14) were included. Patients who were reluctant to participate or had any complication leading to alterations of the anesthesia method, severe spinal deformity, previous back surgery, spinal cord lesions, infection at the site of injection, a history of coagulopathy, an active neurological disease, or a history of allergy to Marcaine or meperidine were excluded.

The patients were recruited through convenience sampling until the desired sample size was achieved. Then, they were randomly assigned to two groups of SA with Marcaine (n = 69) or meperidine (n = 69). Random allocation in Microsoft Excel software was applied to allocate a random even or odd number to each patient. The patient was assigned to the treatment with Marcaine if the number was odd; otherwise, he/she was assigned to treatment with meperidine.

The study was single-blinded, and the person who postoperatively evaluated the patients was blinded to the type of anesthesia. To minimize the potential biases, a target surgeon performed the operations.

### 3.1. Interventions

The patient was seated, and his/her back was rinsed using a betadine solution. Then, a 24-gauge Quincke spinal needle (No. 24) entered the intrathecal space between the fourth and fifth lumbar vertebrae. The excretion of cerebrospinal fluid confirmed the correct entrance to the intrathecal space. The first group was anesthetized using 2.5 mL of hyperbaric Marcaine 0.5%, while the second group was anesthetized with 1 mg/kg of meperidine without any preservative.

The patients remained sitting for another 5 minutes and were eventually positioned in the lithotomy position for the operation.

### 3.2. Outcomes

The demographic information (age, sex, educational level, and employment status) and clinical history (history of anal surgery and the anal sphincter tone at baseline) were recorded in a checklist.

The study’s primary outcome was to evaluate the patients’ pain complaints within 1 day after the surgery. The assessments were made within 2, 4, 6, 12, and 24 hours after the operation using the standard questionnaire of the NRS (14). The patients presented their pain severity under the supervision of a nurse blinded to the type of agent used for anesthesia.

The surgeon recorded the anal sphincter tone using a manometer before the surgery and immediately after the end of the sphincterotomy.

Postoperative pain was managed using intravenous ketorolac (30 mg) (Caspian, Iran); alternatively, within the hospital and afterward, 100 mg of diclofenac tablets (Alborz Darou Company, Iran) was prescribed to consume if needed.

The drug-related complications, including gastrointestinal symptoms (nausea and vomiting), headache, urinary retention, hypotension, and TNS, were evaluated in the two groups.

### 3.3. Statistical Analysis

The data were entered into the Statistical Package for Social Sciences v. 23 (SPSS Inc., Chicago, IL, USA). The qualitative data were presented as absolute numbers and percentages, and the quantitative ones as mean and standard deviation. The normality of the data distribution was assessed using the Kolmogorov-Smirnov test. The categorical data were compared using the chi-square or Fisher’s exact tests. The continuous data were compared using an independent *t*-test. Repeated measures analysis of variance (ANOVA) was applied to compare the trend of changes in the groups. A P-value of less than 0.05 was determined as the significance level.

## 4. Results

**Figure 1.**
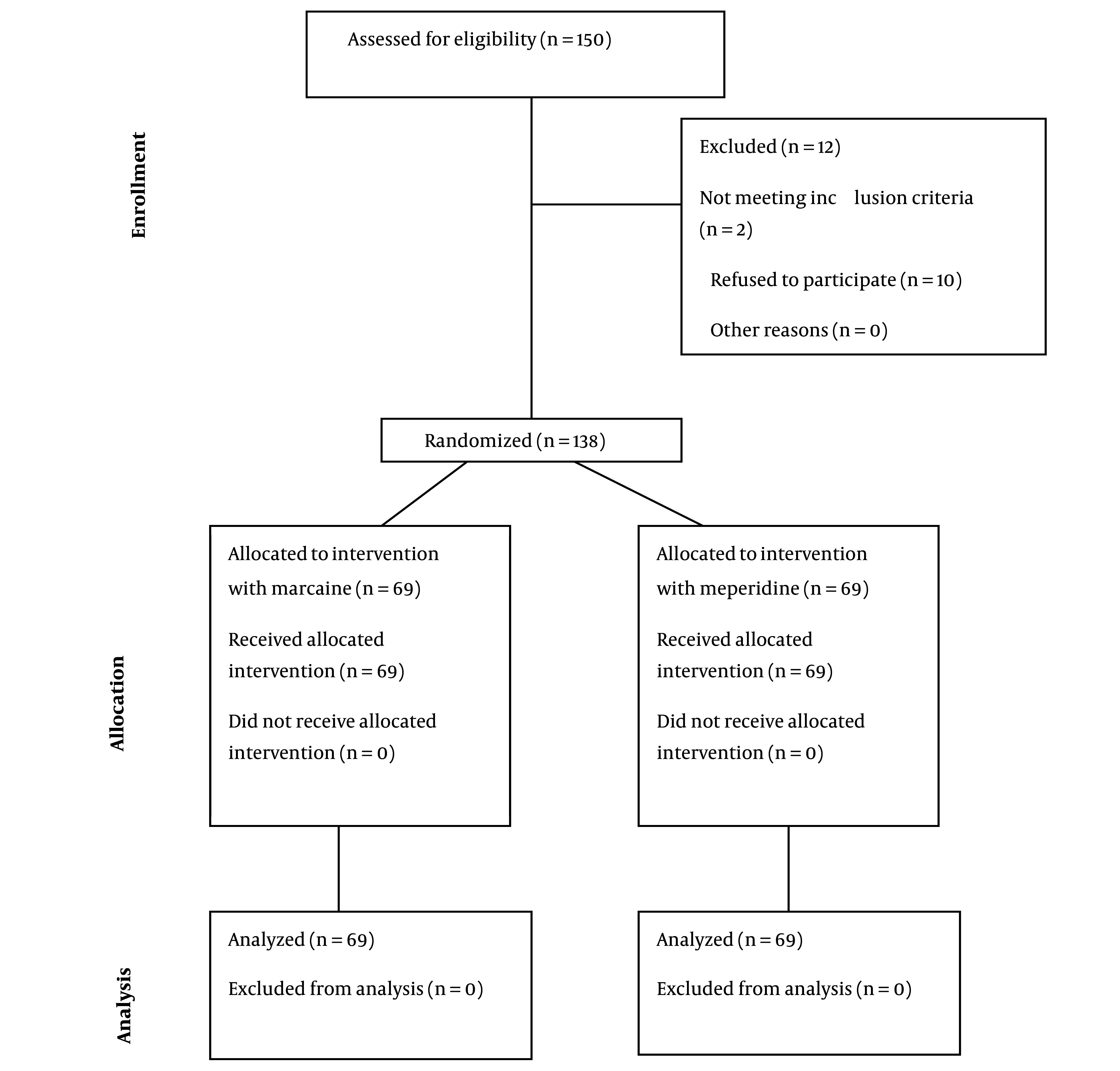
The consort diagram of the population

The data of 150 patients were assessed for eligibility to participate in the study. Of these, 2 patients did not meet the inclusion criteria, and 10 refused to participate. Finally, 138 patients in two equal groups of SA with Marcaine (n = 69) or meperidine (n = 69) were studied (Figure 1). The study population had a mean age of 35.21 ± 7.74 years and predominantly consisted of women (n = 82, 59.42%).

Table 1 demonstrates the demographic and clinical characteristics of the studied population. The two groups were similar in terms of age (P = 0.247), sex distribution (P = 0.119), level of education (P = 0.734), employment status (P = 0.119), a history of anal surgery (P = 0.758), and the baseline sphincter tone (P = 0.119).

**Table 1.** The Demographic and Clinical Characteristics of the Studied Population ^a^

Variables	Meperidine (n = 69)	Marcaine (n = 69)	P-Value
**Age (y)**	35.94 ± 7.7	34.47 ± 7.0	0.247 ^b^
**Sex (female)**	46 (66.7)	36 (52.17)	0.119 ^c^
**Higher education (yes)**	35 (50.72)	34 (49.27)	0.734 ^c^
**Employed (yes)**	36 (52.17)	46 (66.7)	0.119 ^c^
**Previous anal surgery (yes)**	7 (10.14)	8 (11.59)	0.785 ^c^
**Baseline sphincter tone (mmHg)**	76.94 ± 2.91	78.52 ± 4.51	0.119 ^b^

^a^ Values are expressed as mean ± standard deviation or No. (%).

^b^*t*-test

^c^ Chi-square test

No patient complained of pain within 2 hours after the surgery (P > 0.99), while the pain severity was remarkably higher among Marcaine-treated cases in all the assessments (P < 0.05). Besides, the trend of pain complaints declined over time in both groups (P < 0001). Sphincter tone significantly changed after the operation in both groups; however, less tone was noted in the Marcaine-treated cases. The two groups did not differ regarding wound drainage. Table 2 presents detailed data.

**Table 2.** A Comparison of Anesthetic Agents Between the Groups ^a^

Variables	Meperidine (n = 69)	Marcaine (n = 69)	P-Value
**Pain score**			
Baseline	8.25 ± 1.75	8.76 ± 2.02	0.45
Within 2 hours after the surgery	0	0	> 0.99 ^b^
Within 4 hours after the surgery	0	2.55 ± 0.64	< 0.001 ^b^
Within 6 hours after the surgery	2.62 ± 0.87	6.35 ± 1.02	< 0001 ^b^
Within 12 hours after the surgery	6.54 ± 1.32	8.45 ± 2.06	< 0.001 ^b^
Within 24 hours after the surgery	4.22 ± 0.89	6.41 ± 1.12	< 0.001 ^b^
P-value	< 0.001 ^c^	< 0.001 ^b^	
**Sphincter tone (mmHg)**			
Before the surgery	76.94 ± 2.91	78.52 ± 4.51	0.119 ^b^
After the surgery	65.22 ± 3.02	46.04 ± 1.97	< 0.001 ^b^
P-value	< 0.001 ^b^	< 0.001 ^b^	
**Wound drainage**	17 (24.63)	22 (31.88)	0.253 ^d^

^a^ Values are expressed as mean ± standard deviation or No. (%).

^b^*t*-test

^c^ Repeated measures analysis of variance

^d^ Chi-square test

The complications related to the type of agent used for SA represented nonsignificant differences (P > 0.05; Table 3). 

**Table 3.** A Comparison of Drug-Related Side Effects ^a, b^

Variables	Meperidine (n = 69)	Marcaine (n = 69)	P-Value
**Gastrointestinal side effects**	7 (10.14)	7 (10.14)	> 0.99
**Hypotension**	0 (0)	1 (1.44)	0.316
**Urinary retention**	0 (0)	3 (4.34)	0.079
**Headache**	19 (27.53)	20 (28.98)	0.850
**Transient neurologic symptoms**	5 (7.24)	9 (13.04)	0.259

^a^ Values are expressed as No. (%).

^b^ Chi-square test

## 5. Discussion

Due to the potential complications of GA, surgeons have increasingly favored SA for diverse operations such as anal fissure surgery; still, the agent by which optimal outcomes with minimal complications can be achieved is a matter of debate. Along with the adverse effects of anesthetics, the reduction of postoperative pain in the surgical management of anal fissures should be considered (15).

This study evaluated the efficacy and complications of meperidine versus Marcaine for SA induction in anal fissures. We found less severe pain complaints in those treated with meperidine than Marcaine in all the assessments, while a significant deterioration of pain accompanied both approaches 24 hours after the intervention. Both agents significantly improved anal tone manometry; those treated with Marcaine represented sphincter tone of the normal range, but the tone was slightly above the normal range in Marcaine-treated cases. The two agents were similar in terms of complications.

These two agents are among the oldest used for SA induction; nevertheless, limited knowledge is available regarding their administration for anorectal surgeries. In most studies, they have been used with other agents or other types of interventions, particularly gynecological ones (16-19).

Arjumand et al. compared the efficacy and adverse effects of meperidine versus Marcaine for SA in the surgical management of various surgeries (20). They administered 2.5 mL of isobaric 0.5% bupivacaine or 1 mg/kg of preservative-free pethidine and found insignificant differences between the groups regarding postoperative pain complaints and complications; however, a more rapid recovery profile was noted among those receiving pethidine (20). Another study in a similar context was published by Udonquak et al., who applied meperidine at a dose of 1 mg/kg and compared it with 2.5 mL 0.5% (21). While complications, including urinary retention, were significantly higher among those receiving Marcaine, the latter groups complained of pruritis remarkably more. The two groups were similar in terms of nausea and vomiting incidence. Their study culminated in similar postoperative pain severity; however, an earlier recovery profile outweighed meperidine (21).

Aminisaman and Hasani investigated various parameters, including the duration of anesthesia and analgesia, hemodynamic changes, and complications after SA induced by 12.5 mg of bupivacaine 0.5% (2.5 mL) versus 1 mg/kg of preservative-free pethidine in older patients; however, they did not limit the type of surgery for which the patients received SA (22). They evaluated 66 patients aged over 60 years old. In agreement with our findings, they reported that given the different aspects of opioid use, it seems pethidine is more efficient due to a longer analgesic time, similar hemodynamic changes, fewer headaches, and less occurrence of shivering compared to bupivacaine in elderly patients (22).

Similar outcomes in favor of meperidine were reported by Forouzesh Fard et al., who presented reasonable postoperative pain control with negligible adverse effects and acceptable labor outcomes among women who received SA to deliver their child through cesarean section (23).

Numerous recent studies have assessed the intrathecal administration of Marcaine and compared it with other agents, particularly mepivacaine, for SA induction among those undergoing total hip arthroplasty. These studies found promising outcomes for both drugs; still, earlier ambulation marked the superiority of mepivacaine over Marcaine (24-27).

Transient neurologic symptoms are the most significant adverse effect of this group of agents, including Marcaine, prilocaine, mepivacaine, procaine, ropivacaine, levobupivacaine, and 2-chloroprocaine, regardless of their baricity (28). The other complication of Marcaine is urinary retention which might limit preferences for its use, as mentioned in various investigations (24-27).

Morphine is the most popular narcotic analgesic for pain management and SA in diverse conditions. Information about SA for anorectal surgeries using narcotics is limited, whereas Moreira declared promising outcomes for the subarachnoid injection of morphine for anal fissure operation; still, urinary retention and cutaneous pruritis were the most significant complications (11). Meperidine is another agent with a similar biological action but one-tenth of its potency. This agent has rarely been investigated for SA in anorectal operation, but some investigations have favored it and presented the mentioned adverse effects as the limitations (29). The probability of hemodynamic instability with a sudden reduction in heart rate and blood pressure is the other side effect of meperidine that should be considered (30, 31).

In summary, we found the superiority of meperidine over Marcaine for SA in patients undergoing anal fissure surgical management. For the first time, this study evaluated the significance of anal sphincter tone for the early return of the bowel system to normal functioning and as a contributor to pain severity. A sphincter tone decline to normal values accompanied Marcaine’s use. While the body of evidence has focused chiefly on the agents applied for SA rather than using them for anorectal interventions, the novelty and strength of our investigation lie in its dedication to anorectal surgeries. Accordingly, further investigations on this topic are recommended.

### 5.1. Limitations

The small sample size and the short follow-up period were the most significant limitations of our study. Besides, some of the variables affecting the response to the treatment, such as the period of suffering from the anal fissure or the patients’ routine daily regimen that could affect their bowel habits, were not studied. Another significant limitation is the failure to assess the amount of analgesics used by the patients in the postsurgical setting to control their pain. This factor could have significantly affected their NRS scores.

### 5.2. Conclusions

Meperidine used for SA in anal fissure surgical management was relatively superior to Marcaine as postoperative pain control was remarkably better achieved. The anal sphincter tone returned to the normal range in Marcaine-treated cases, whereas those anesthetized with meperidine had an average tone slightly above the normal limits. Further evaluations with diverse doses of the drugs are strongly recommended.

## Data Availability

The dataset presented in the study is available upon request from the corresponding author during submission or after publication. The data are not publicly available due to further investigations being in progress on these data.

## Clinical Trial Registration:

IRCT20180921041078N1.
